# Solidification Behavior of Fe-6.5Si Alloy Powder for AM-SLM Processing, as Assessed by Differential Scanning Calorimetry

**DOI:** 10.3390/ma16124229

**Published:** 2023-06-07

**Authors:** Darja Steiner Petrovič, Črtomir Donik, Irena Paulin, Matjaž Godec, Maja Vončina, Martin Petrun

**Affiliations:** 1 Institute of Metals and Technology, Lepi Pot 11, 1000 Ljubljana, Slovenia; crtomir.donik@imt.si (Č.D.); irena.paulin@imt.si (I.P.); matjaz.godec@imt.si (M.G.); 2 Faculty of Natural Sciences and Engineering, University of Ljubljana, Aškerčeva 12, 1000 Ljubljana, Slovenia; maja.voncina@ntf.uni-lj.si; 3 Faculty of Electrical Engineering and Computer Science, University of Maribor, Koroška 46, 2000 Maribor, Slovenia; martin.petrun@um.si

**Keywords:** Fe-6.5wt%Si powder, soft magnetic alloy, differential scanning calorimetry, oxygen, high-temperature phase, microstructure, eutectics

## Abstract

Lab-scale investigations on the processing of small powder volumes are of special importance for applications in additive manufacturing (AM) techniques. Due to the technological importance of high-silicon electrical steel, and the increasing need for optimal near-net-shape AM processing, the aim of this study was to investigate the thermal behavior of a high-alloy Fe-Si powder for AM. An Fe-6.5wt%Si spherical powder was characterized using chemical, metallographic, and thermal analyses. Before thermal processing, the surface oxidation of the as-received powder particles was observed by metallography and confirmed by microanalysis (FE-SEM/EDS). The melting, as well as the solidification behavior of the powder, was evaluated using differential scanning calorimetry (DSC). Due to the remelting of the powder, a significant loss of silicon occurred. The morphology and microstructure analyses of the solidified Fe-6.5wt%Si revealed the formation of needle-shaped eutectics in a ferrite matrix. The presence of a high-temperature phase of silica was confirmed by the Scheil–Gulliver solidification model for the ternary model Fe-6.5wt%Si-1.0wt%O alloy. In contrast, for the binary model Fe-6.5wt%Si alloy, thermodynamic calculations predict the solidification exclusively with the precipitation of b.c.c. ferrite. The presence of high-temperature eutectics of silica in the microstructure is a significant weakness for the efficiency of the magnetization processes of soft magnetic materials from the Fe-Si alloy system.

## 1. Introduction

For the electromagnetic properties of Fe-Si soft magnetic alloys to be optimal, silicon content of 6.5 wt.% is the most effective. This level of silicon concentration ensures near-zero magnetostriction, low electric losses, high permeability, and high electrical resistivity [1,2,3,4]. However, it should be noted that such a high silicon concentration can result in the material becoming brittle and susceptible to embrittlement due to the formation of ordered phases [5,6,7,8,9,10]. Fortunately, rapid cooling can prevent the formation of these ordered phases. It is important to consider the cooling rate, as this affects the solidification temperature profile, which subsequently impacts the ordering, microstructure, hardness, and magnetic properties of the material.

A challenge that needs to be overcome is how to process magnetic materials and components that contain 6.5 wt% Si (11 at% Si) beyond the current technological routes [1,2,3,4,5,6,7,8,9,10]. With growing interest in electrification based on clean-energy technologies, the demand for next-generation high-performance magnetic materials has expanded a great deal [6,7,8,9,10,11,12,13,14].

Over the past few years, metal AM has been growing remarkably. AM, a layer-by-layer three-dimensional (3D) printing technology, has opened up new possibilities for improvements in the industrial manufacturing of electrical machines via the near-net-shape printing of complex geometries, tailored structure–composition–property relationships, a reduction in the part count and the production lead time, and the conservation of expensive critical raw materials [6,15,16,17,18,19].

The AM of ferromagnetic alloys has received much attention because of its potential to produce functional parts [19,20,21]. Paramagnetic powders have also been used for the production of gradient soft magnetic materials with different structural and physical properties produced by an AM technique using in situ melting during direct-energy deposition [22]. Successful combinations of AM and topology optimization for fabricating both the iron core and the permanent magnet with the desired performance show great potential as a tool for the designers of electrical machines [17].

Metal AM can use different forms of feedstock, with powders and wires being the most common. The selection of feedstock type has a great impact on (i) the overall cost of the process, (ii) the print speed and resolution, and (iii) the quality and safety [19].

An important shortcoming of AM techniques is the cracks that appear very frequently [23]. The heterogeneity and high dynamics of the temperature fields that are characteristic for the AM technique, as well as the transfer of substances due to Marangoni convection, contribute to the significant segregation of elements and the formation precipitate phases. One of the reasons for crack formation is the presence of Fe_3_C in the area of ordered phases [23]. However, by means of electron beam powder bed fusion (E-PBF), crack-free Fe-Si soft magnetic materials have already been successfully manufactured [24].

Despite its potential benefits, AM still presents several challenges, including expensive processing costs, post-processing difficulties, and supply chain maturity issues [19,20,21,22,23,24]. Many open research questions need to be addressed to optimize the use of AM in electrical machines, such as the feasibility of using multi-material 3D printing to create machine parts with excellent electromagnetic performance, such as winding with insulation or core, as well as the development of new soft magnetic materials or alloys with high magnetic permeability and low losses, such as Fe-Si powders or Fe-Co [19].

Numerous studies have been conducted on alloys, but unfortunately, many of them lack crucial details on the mechanical and magnetic properties of these materials. These properties are of significant importance when it comes to understanding how alloys perform under different conditions. Thus, it is essential to conduct further research and tests on various alloy systems, particularly the emerging advanced alloys and composites found in the complex concentrated alloy or high-entropy alloy space. It is also crucial to test integrated electromagnetic machine components that utilize magnetic cores enabled by unconventional processing techniques. By doing so, researchers can gain a better understanding of how these alloys and components can be optimized for maximum performance [6].

At present, the metallurgical market offers electrical steel, a product that is commonly a sheet or strip with a silicon content of less than 4 wt%. However, with the advances in modern electrical machinery, there is a growing demand for high-silicon Fe-Si alloys that exhibit exceptional magnetic properties. As a result, the present study focuses on improving the processability of high-alloy Fe-Si powder and, consequently, the magnetic characteristics of such alloys.

Due to the technical importance of Fe-Si alloys, the study involved analysing the thermal behaviour of an Fe-6.5wt% Si alloy powder through simultaneous thermal analysis. By doing so, the researchers were able to gain a better understanding of the thermal properties of the alloy and identify areas where improvements could be made. Ultimately, the aim of this research is to support the development of high-performance Fe-Si alloys that meet the specific requirements of modern electrical machinery.

## 2. Material and Methods

### 2.1. Sampling

For representative powder particles sampling, the simple random sample method was chosen. Two hypotheses were set: (i) each unit in the population has an equal possibility of being selected and (ii) the sample properties are a true reflection of the population’s properties [25].

Samples of Fe-6.5wt%Si alloy powder were further investigated by means of chemical analyses, metallography, and thermal analysis. For chemical analyses, a sample mass of 1 g was taken. For the FE-SEM/EDS metallographic examination, samples were taken randomly using carbon tape. For thermal analysis, a sample mass of 1 g was taken.

Sampling from a population with an arbitrary distribution of the attribute was conducted to determine the silicon content in the selected samples of the DSC-remelted bulk of an Fe-6.5wt%Si alloy. Here, the elements of a population are characterized by a measurable attribute (e.g., by concentration) [25]. For chemical analyses, a sample mass of 1 g was taken.

### 2.2. Chemical Analyses

The chemical compositions of the randomly selected samples (of 1 g in weight) were determined using an optical emission spectrometer with inductively coupled plasma (OES-ICP, Agilent Technologies, Inc., Santa Clara, CA, USA), an Agilent 5800 VDV instrument (Agilent Technologies, Inc., Santa Clara, CA, USA), and combustion methods using an ELTRA CS-800 analyser for carbon and sulphur estimation (ELTRA, GmbH, Haan, Germany). Furthermore, Si content in the as-received powdered alloy samples and the remelted Fe-6.5wt%Si bulk samples was additionally determined by gravimetric and classical analytical methods.

### 2.3. Metallography

Metallographic analyses FE-SEM/EDS were performed using a scanning electron microscope JEOL JSM-6500F (JEOL Ltd., Tokyo, Japan). FE-SEM/EDS analyses of the powder particles were performed at an accelerating voltage of 15 kV.

Optical imaging was performed using a ZEISS Axio Imager Z2M (Carl Zeiss AG, Oberkochen, Germany) optical microscope.

Samples under investigation were randomly selected powder particles and metallographic cross-sections of the DSC-remelted bulk samples of Fe-6.5wt%Si.

### 2.4. Thermodynamic Calculations

Thermodynamic calculations were performed using the Thermo-Calc (Thermo-Calc Software AB, Solna, Sweden) software package 2017a and the database TCFE8:Steels/Fe-Alloys v8.1. A computer simulation of the solidification of the selected binary Fe-6.5wt%Si alloy was performed with the Scheil–Gulliver model [26]. For comparison, the solidification path of a model alloy, an oxidized ternary Fe-6.5wt%Si-1.0wt%O alloy, was also simulated.

### 2.5. Thermal Analysis (DSC)

The thermal behavior of the soft magnetic alloy powder for AM was investigated using differential scanning calorimetry (DSC) with a STA-449 C Jupiter instrument, Netzsch (Netzsch-Gruppe, Selb, Germany).

The dynamic measurements involved two consecutive cycles at the same selected ramp rate, i.e., 10 K/min. The linear temperature program for heating and cooling was as follows: 25 °C–1550 °C–350 °C–1550 °C–25 °C. A regular temperature calibration was conducted using technically pure metals at 10 K/min as a medium scanning rate using an argon atmosphere (Ar, 5N). For a low-temperature region, In, Sn, and Al were used. In the case of the high-temperature region, the calibration involved Au and Ni. Measurements of temperature were made using S-type thermocouples (for the furnace as well as the sample carrier control). Accordingly, the DSC experiments were conducted under a static argon atmosphere. There was no isothermal annealing performed at the maximum temperature. Two cycles were used to achieve better thermal contact between the two forms of specimens (powder particles and remelted alloy) and the crucible.

DSC experiments were conducted in three parallels; randomly selected samples (1 g in weight) were designated as #1, #2, #3.

## 3. Results and Discussion

### 3.1. Chemical Analysis of Alloy Powder

The chemical compositions of the selected samples of Fe-6.5wt%Si alloy powder are shown in Table 1. A low carbon content is important to prevent magnetic aging [27].

### 3.2. SEM/EDS Analyses of Alloy Powder

**Figure 1:**
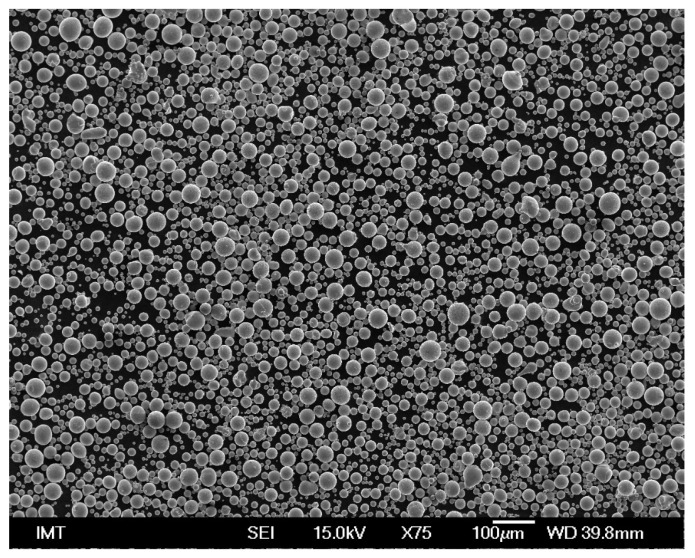
Morphology of the Fe-6.5wt%Si alloy powder aimed for AM.

Figure 1 shows that particles of the alloy powder designed for the AM are spheres. Their mean diameter is 45 µm.

**Figure 2:**
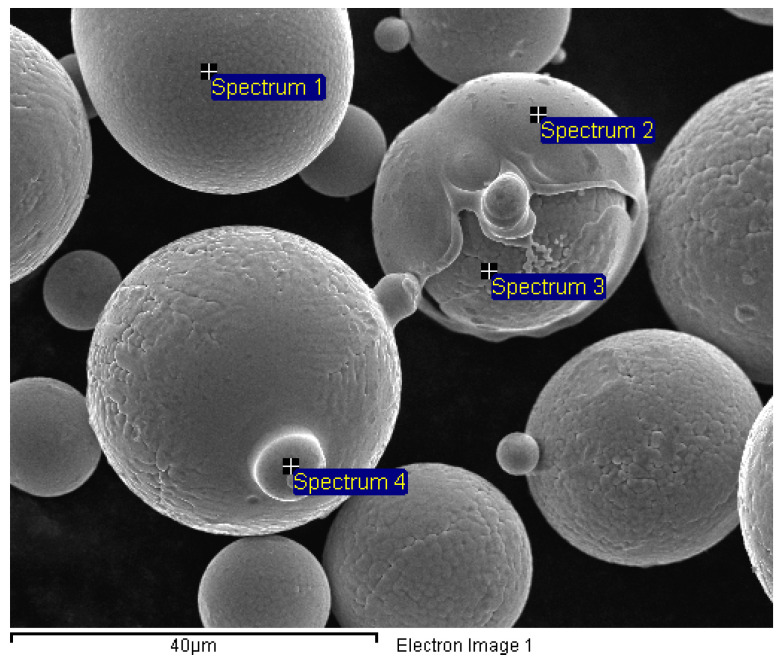
Oxidized surface of the Fe-6.5wt%Si alloy powder.

Further characterization of the Fe-6.5wt%Si powder using SEM/EDS revealed oxidized surfaces of the randomly selected metal particles (Figure 2). Information on the relative concentrations of the elements present in the analyzed metal particles from Figure 2 can be found in Table 2.

The measurements show that the concentrations of powder particles, specifically the Si content, vary in relative amounts. However, it is essential to focus on the final concentration of the prepared material rather than the precursors used. The uneven distribution of the element in the sphere and the teardrop-shaped interaction volume of the EDS can cause variations in the measured values. At the 15 kV accelerating voltage, the EDS can penetrate the surface from less than 100 nm up to a depth of 2 μm. The theoretical detection limits in SEM–EDS measurements are about 0.1 wt.%.

### 3.3. Thermodynamic Calculations

Due to the SEM-EDS results showing oxidized surfaces of the Fe-6.5wt%Si alloy powder, TD equilibrium and Scheil–Gulliver solidification modelling were performed for two alloys: the binary model Fe-6.5wt%Si alloy and the ternary model Fe-6.5wt%Si-1.0wt%O alloy.

**Figure 3:**
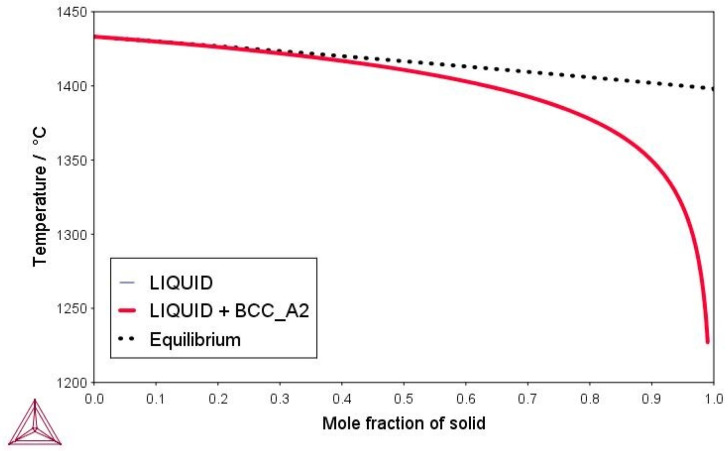
Scheil–Gulliver solidification model of the binary model Fe-6.5wt%Si alloy.

**Figure 4:**
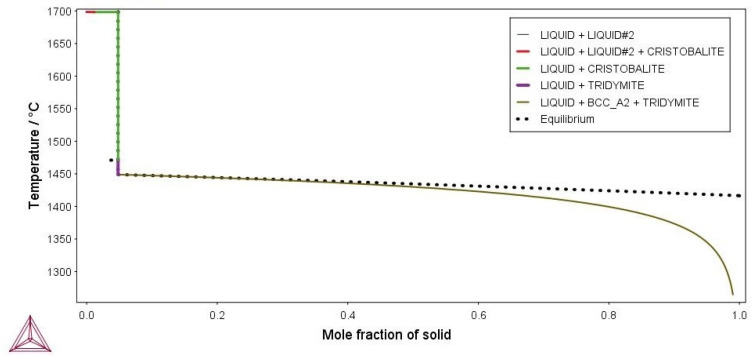
Scheil–Gulliver solidification model of the ternary model Fe-6.5wt%Si-1.0wt%O alloy.

The simulations are graphically shown in Figure 3 and Figure 4. The model binary Fe-6.5wt%Si alloy solidifies with the formation of b.c.c. ferrite (see Figure 3). In the ternary Fe-6.5wt%Si-1.0wt%O alloy, cristobalite primarily precipitates from the liquid, solidification proceeds with the precipitation of tridymite, and finally the alloy solidifies with the precipitation of b.c.c. ferrite. Cristobalite and tridymite are high-temperature polymorphs of silica, forming stably above 870 °C (tridymite) and 1470 °C (cristobalite) (see Figure 4).

### 3.4. Thermal Analysis (DSC)

In the DSC experiments, the samples of the soft magnetic Fe-6.5wt%Si alloy powder were linearly heated at 10 K/min above the liquidus temperature to allow their melting behavior to be monitored. Cooling at the same ramp rate allowed the solidification sequence to be monitored. Linear heating and cooling at the selected ramp rate were repeated in two consecutive cycles.

**Figure 5:**
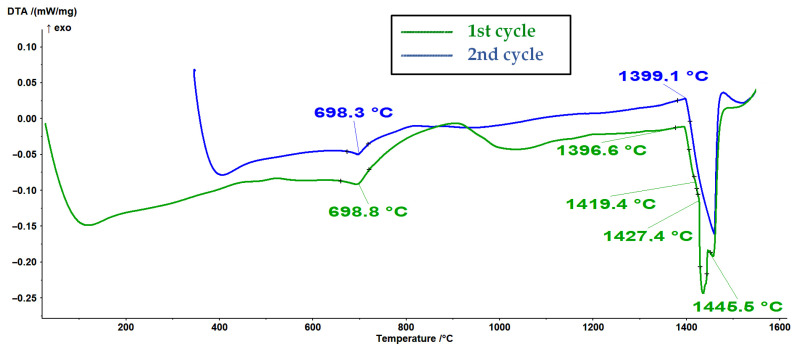
DSC melting curves of sample #1 of the Fe-6.5Si powder (1st and 2nd cycles, with a heating rate of 10 K/min).

**Figure 6:**
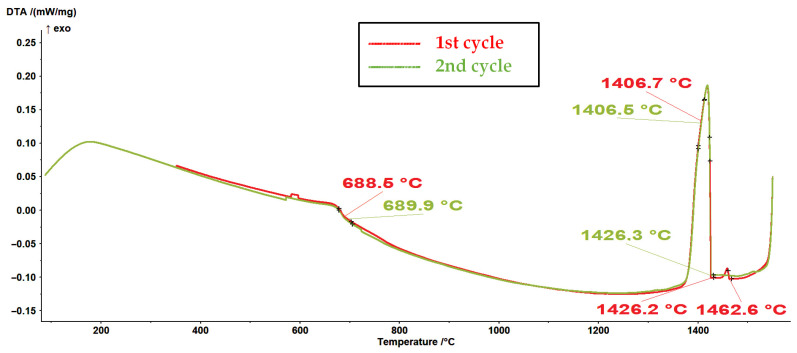
DSC cooling curves of the #1 sample of the Fe-6.5Si powder (1st and 2nd cycles, with a heating rate of 10 K/min).

In Figure 5 and Figure 6, the first and second cycles of the DSC experiments on melting and cooling, respectively, are represented for the powder sample designated as #1. As can be seen in Figure 5, the reactions in the melting DSC experiments are characterized by endothermic peaks, whereas the reactions in the DSC cooling experiments are characterized by the presence of exothermic peaks (Figure 6).

**Figure 7:**
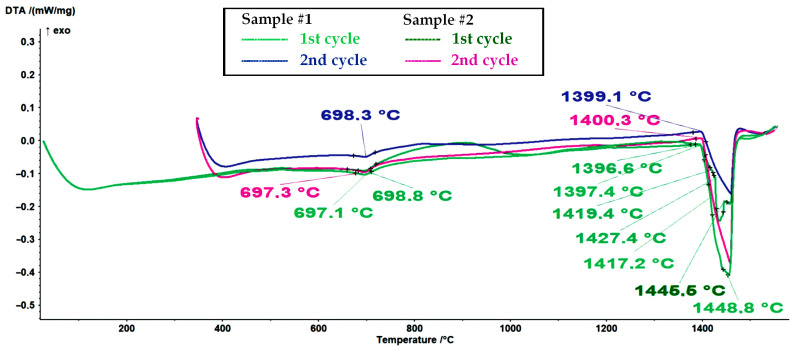
A comparison of DSC melting curves of sample #1 and a non-oxidized sample #2 (1st and 2nd cycles, with a heating rate of 10 K/min).

**Figure 8:**
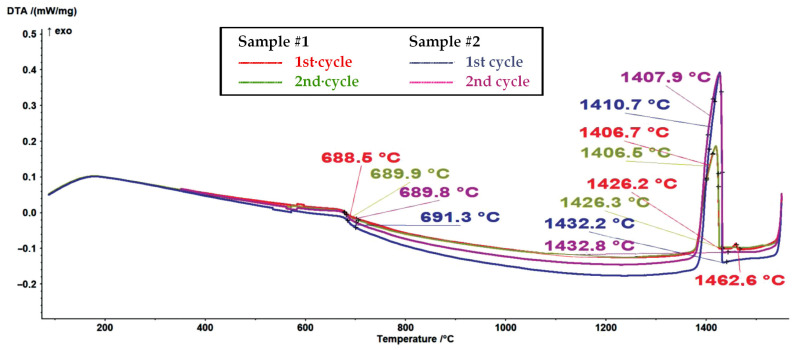
A comparison of DSC cooling curves for sample #1 and a non-oxidized sample #2 (1st and 2nd cycles, with a heating rate of 10 K/min).

It is assumed that the first endothermic peak at approximately 698 °C in the DSC melting curves (see Figure 5) represents the ferromagnetic-to-paramagnetic transition, i.e., the Curie transition. Analogous to this, the first exothermic peak at approximately 689 °C in the DSC curves from Figure 6 represents the Curie transition in the material on cooling. The results (see Figure 5, Figure 6, Figure 7 and Figure 8) are in very good agreement with the study on the solid solubility of Si in sintered Fe–Si alloys performed by Yuan et al., who showed that the DSC method can also be used as a reliable technique to characterize the evolution of the solid solubility of Si in iron [28].

Even more interesting was the DSC cooling curve for sample #1, represented in Figure 6 and Figure 8, where an additional exothermic peak could be observed with an onset temperature of 1462.6 °C.

It is clear that sample #1 contained a fraction of unknown high-temperature phase(s). When an alloy contains only a small fraction of the high-temperature phase, determining the liquidus temperature in DTA and DSC measurements can be very challenging [29]. However, based on the thermodynamic calculations for a ternary model alloy from the alloy system Fe-Si-O, it can be assumed that the high-temperature phases are high-temperature polymorph(s) of silica (see Figure 4 and Table 3).

Since the second cycle of the DSC experiments did not show the presence of high-temperature phase(s), we can conclude that the surface-oxidized alloy powder was sufficiently degassed (deoxidized) during the processing stage in an Ar atmosphere (processing via melting–cooling–remelting–cooling).

**Figure 9:**
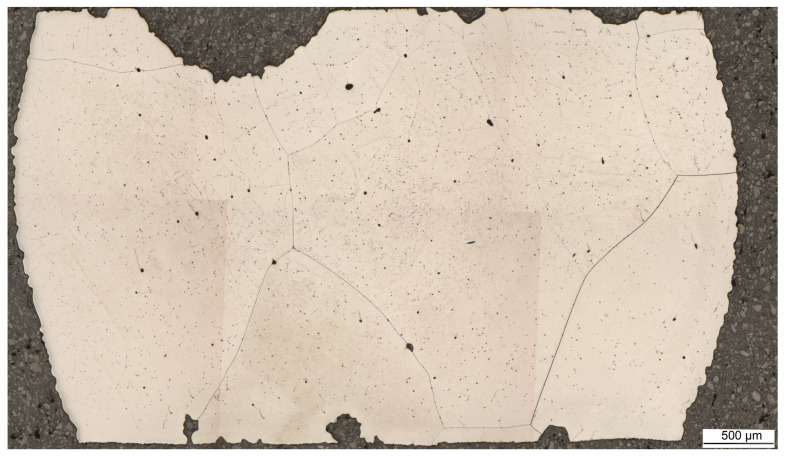
Typical ferrite microstructure of the remelted and solidified Fe-6.5Si alloy powder—sample cross-section (LM, mag. 50×. Etchant: Nital).

In Table 3, the characteristic temperatures of primary precipitation during solidification are listed. The solidification process for the Fe-6.5wt%Si alloy powder was also assessed using light microscopy. Figure 9 shows the solidified microstructure of the Fe-6.5wt%Si alloy powder after the DSC experiment.

**Figure 10:**
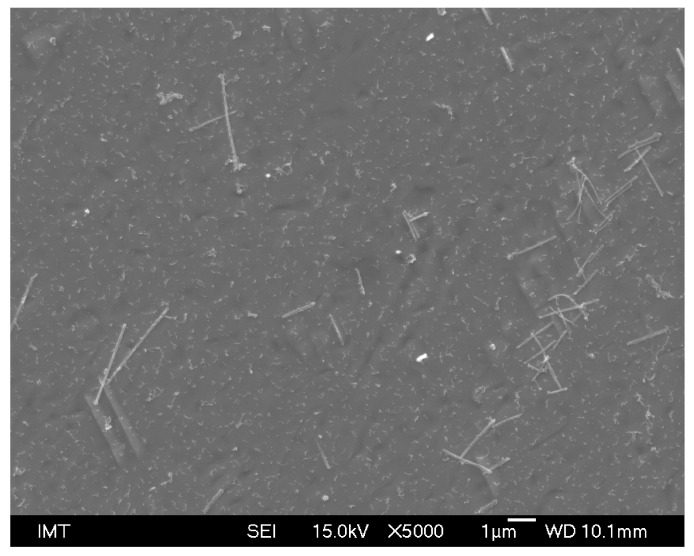
Needle-shaped eutectic in the matrix of remelted and solidified Fe-6.5wt%Si alloy powder (SEM, mag. 5000×).

Due to the rather slow cooling rate and very low impurity content, especially of carbon, the typical remelted and solidified microstructure consists of large polygonal ferrite grains, where the diameter of individual grains reaches up to several millimeters. Shrinkage porosity visible in the form of large, black, round spots and many small precipitates are present. However, a detailed look at the solidification microstructure reveals the obvious substructures in the underlying ferrite matrix (Figure 10). Furthermore, in the ferrite matrix, needle-shaped eutectic structures can be observed as well (Figure 10).

In addition, after the DSC processing of the soft magnetic Fe-6.5wt%Si powder alloy, chemical analysis revealed a noticeable silicon loss (>5%). The results are listed in Table 4.

A significant evaporation of Si during annealing was also reported in the study of Tian et al., where Fe-6.5wt%Si sheets were prepared based on various processes, including magnetron sputtering [1].

The metallographic characterization of the Fe-6.5wt%Si powder revealed the surface oxidation of spherical particles (Figure 2). Both the high affinity of Si for oxygen and the large surface area of the powder resulted in the formation of a high-temperature eutectic during remelting. Therefore, the presence of high-temperature phases from the Fe-Si-O alloy system is assumed.

The solidification microstructures of the model Fe-6.5wt%Si alloy, the oxidized Fe-6.5wt%Si alloy powder, and the conventional high-silicon electrical steels are certainly comparable.

The solidification microstructure of all of the selected alloys consists of a b.c.c.-ferrite matrix. However, the microstructure of the model Fe-6.5wt%Si alloy is fully ferritic [30], whereas the microstructure of the fully processed conventional high-silicon electrical steels is composed of a b.c.c.-ferrite matrix with some non-metallic inclusions (NMIs) present as well [27].

The presence of high-temperature oxides or eutectics is definitely not the case in conventional high-silicon electrical steels [31], as observed in the remelted and solidified oxidized Fe-6.5wt%Si alloy powder. This is a significant weakness of the efficiency of the magnetization processes for soft magnetic materials from the Fe-Si alloy system.

## 4. Conclusions

The experiments showed that the chemical compositions of the analyzed samples of the Fe-6.5wt%Si alloy powder differ from each other.

According to relevant TD calculations, the model binary Fe-6.5wt%Si alloy solidifies with the primary form of b.c.c. ferrite.

However, during the solidification of some Fe-6.5wt%Si alloy powder under investigation, the DSC measurements revealed the precipitation of not only b.c.c. ferrite, but also of a small fraction of high-temperature phase(s).

Due to the high affinity of Si for oxygen, and the large surface area of the alloy powder, it can be concluded that the high-temperature phases are silica polymorphs.

Metallographic analyses on the remelted and solidified Fe-6.5wt%Si alloy powder revealed the formation of needle-shaped eutectics in the ferritic matrix.

In addition, a significant loss of silicon through remelting was measured.

## Figures and Tables

**Table 1:** Chemical compositions of the Fe-6.5wt%Si alloy powders (wt. %).

Element	Sample Powder #1	Sample Powder #2	Sample Powder #3
Si	6.63 ± 0.02	6.63 ± 0.02	6.66 ± 0.02
C	0.005 ± 0.0003	0.005 ± 0.0003	0.005 ± 0.0003
S	0.004 ± 0.0002	0.005 ± 0.0002	0.004 ± 0.0002
Fe	balance	balance	balance

**Table 2:** EDS point analysis results of metal particles from Figure 2 (in wt.%).

Spectrum	O	Si	Fe
Spectrum 1	0.6 ± 0.03	8.1 ± 0.4	91 ± 2
Spectrum 2	1.3 ± 0.05	2.5 ± 0.1	96 ± 3
Spectrum 3	2.1 ± 0.09	9.7 ± 0.5	88 ± 2
Spectrum 4	1.8 ± 0.09	7.7 ± 0.4	90 ± 2

**Table 3:** A comparison of characteristic temperatures of precipitation on cooling—modelling vs. experimental results (temperatures in °C).

	Precipitation High-*T* Phase		Precipitation α_prim_
TD Calculations for Model Alloys (Scheil-Gulliver)			
Fe-6.5wt%Si		*	1433.5
Fe-6.5wt%Si-1.0wt%O	Cristobalite Trydimite	1695 1470.8	1448.5
Experimental results for Fe-6.5wt%Si alloy powder			
DSC #1			
1st cooling curve	1462.6		1426.2
2nd cooling curve	*		1426.3
DSC #2			
1st cooling curve	*		1432.2
2nd cooling curve	*		1432.8

* not observed under applied conditions.

**Table 4:** Average silicon content in the Fe-6.5wt%Si alloy powder and in alloys after DSC processing (wt. %).

Alloy Powder	Remelted Alloy #1	Remelted Alloy #2	Remelted Alloy #3
6.6 ± 0.02	6.1 ± 0.02	6.2 ± 0.02	6.1 ± 0.02

## Data Availability

The data used to support the findings of this study are available upon request.
